# Peptides interfering with protein-protein interactions in the ethylene signaling pathway delay tomato fruit ripening

**DOI:** 10.1038/srep30634

**Published:** 2016-08-01

**Authors:** Melanie M. A. Bisson, Mareike Kessenbrock, Lena Müller, Alexander Hofmann, Florian Schmitz, Simona M. Cristescu, Georg Groth

**Affiliations:** 1 Biochemical Plant Physiology, Heinrich-Heine-University Düsseldorf, D-40204 Düsseldorf, Germany; 2 Department of Molecular and Laser Physics, Radboud University, Heyendaalseweg 135, 6525 AJ Nijmegen, The Netherlands

## Abstract

The plant hormone ethylene is involved in the regulation of several processes with high importance for agricultural applications, e.g. ripening, aging and senescence. Previous work in our group has identified a small peptide (NOP-1) derived from the nuclear localization signal of the *Arabidopsis* ethylene regulator ETHYLENE INSENSITIVE-2 (EIN2) C-terminal part as efficient inhibitor of ethylene responses. Here, we show that NOP-1 is also able to efficiently disrupt EIN2-ETR1 complex formation in tomato, indicating that the NOP-1 inhibition mode is conserved across plant species. Surface application of NOP-1 on green tomato fruits delays ripening similar to known inhibitors of ethylene perception (MCP) and ethylene biosynthesis (AVG). Fruits treated with NOP-1 showed similar ethylene production as untreated controls underlining that NOP-1 blocks ethylene signaling by targeting an essential interaction in this pathway, while having no effect on ethylene biosynthesis.

Recent studies on global food losses and food waste indicate that 30–40% of the food produced on earth goes to waste before it can be consumed^1^. Reasons for post-harvest losses are related to developmental (germination, ripening, wilting, senescence), pathological (fungal or bacterial infection) and physical (mechanical injury) processes. Of these processes ripening, aging and senescence are promoted by the plant hormone ethylene, which is produced essentially in all parts of higher plants, including leaves, stems, roots, flowers, fruits, tubers and seeds. Moreover, ethylene is also known to be involved in stress-related responses such as pathogen defense and wounding^2^,^3^. Biosynthesis and signal transduction of the plant hormone have been studied in great detail in the small crucifer weed *Arabidopsis thaliana* and many elements of both pathways have been identified in these studies^4^. The ethylene signal is perceived by a family of five receptor proteins^5^, which form homo- and heterodimers at the ER-membrane^6^. The receptor proteins form ER-borne complexes with the protein kinase CONSTITUTIVE TRIPLE RESPONSE-1 (CTR1)^7^,^8^ and the integral membrane protein EIN2 9,10,11, allowing phosphorylation of EIN2 by CTR1. In the presence of ethylene CTR1 is inactivated leading to dephosphorylation of EIN2 12. As a consequence, the C-terminal domain of EIN2, containing a highly conserved nuclear localization signal (NLS)^13^,^14^, is cleaved by a so far unknown protease and translocated to the nucleus^12^,^14^,^15^. In the nucleus, the EIN2 C-terminus directly or indirectly stabilizes the transcription factor EIN3 15,16 and transcription of ethylene response genes is activated. In addition to its nuclear effects, the C-terminal domain of EIN2 was shown to affect ethylene responses by inhibiting *EBF1/2* mRNA translation and recruiting these transcripts to cytoplasmic P-bodies^16^,^17^.

In the past, various approaches to delay fruit ripening and senescence have been developed. In addition to storage and transport of vegetables and fruits at low temperatures and modified atmosphere with nitrogen and carbon dioxide, these approaches involve inhibition of ethylene biosynthesis, inhibition of ethylene perception or inhibition of ethylene-induced target proteins. Inhibition of ethylene biosynthesis in plants and the related delay in fruit ripening is obtained either by inactivation of ethylene biosynthesis genes in transgenic plants^18^ or by chemicals such as Co^2+^, aminoethoxyvinylglycine (AVG) or aminooxyacetic acid that interfere with ethylene biosynthesis^19^,^20^. Inhibition of ethylene perception is achieved by genetic modification of receptors in transgenic plants^21^ or by application of ethylene antagonists such as carbon monoxide, isothiocyanates, alkenes or alkene-related compounds^22^. Silver nitrate and silver thiosulfate are also effective ethylene antagonists. However, because of their toxicity, their use remains limited to cut flowers. A common characteristic of all chemicals targeting ethylene perception except for silver salts–where silver(I) ion substitutes for the copper-cofactor essential for ethylene binding–is that they are difficult to handle due to their gaseous and/or hydrophobic nature and due to their low water solubility. Moreover, in many cases, mainly in European countries, these chemicals cannot be used because of lack of regulatory approval. Besides, their application often requires close control of treatment methods for food safety reasons. Inhibition of ethylene-induced target proteins promoting fruit softening, accumulation of sugars, acids, pigments, and release of volatiles is restricted to cell wall modifying enzymes such as polygalacturonase or pectin methylesterase in transgenic plants^23^,^24^,^25^.

Recent studies in our lab uncovered a novel way to interfere with ethylene signaling and demonstrated that the NLS motif (LKRYKRRL) of *Arabidopsis* EIN2 enables tight interaction of EIN2 with the receptors sensing the ethylene signal. A small peptide mimicking the NLS motif was shown to interfere with this interaction^26^. This peptide (NOP-1) deduced from the *Arabidopsis* EIN2 protein also efficiently reduced fruit ripening in tomato. As recent studies on the evolution of ethylene as a plant hormone suggest strong conservation of the elements involved in ethylene signaling^27^,^28^, we propose that reduction of fruit ripening in tomato can be explained by the inhibition of ethylene responses, i.e. the same molecular mechanism as shown for *Arabidopsis*. This hypothesis is supported by the high sequence similarity of ethylene signaling components from *Arabidopsis* and tomato. The essential C-terminal part of EIN2 containing the NLS motif which is cleaved and translocated to the nucleus in response to ethylene^12^,^14^,^15^, is highly conserved across plant species. For *Arabidopsis* and tomato the sequence of the NLS motif is fully conserved (100% identity)^14^,^29^, while the overall sequence similarity between AtEIN2 and LeEIN2 is 65% (Supplementary Fig. S1). Receptor homologs from *Arabidopsis* (AtETR1) and tomato (LeETR1) share 81% sequence identity and 90% sequence similarity^30^,^31^.

In this paper we show that the mode of action of the inhibitory peptide is the same in the weed *Arabidopsis* and in the crop tomato. In addition, we describe the mode of inhibition in a more detailed manner and support the high potential of this small peptide to serve as efficient inhibitor of ethylene responses *in planta* at concentrations similar to other known inhibitors of ethylene perception and biosynthesis.

## Results

### Tomato EIN2 and ETR1 form a tight signaling complex

In order to analyze tomato EIN2-ETR1 complex formation *in vitro*, the C-terminal part of tomato EIN2 (LeEIN2^462–1316^) was cloned, heterologously expressed in *E. coli* and purified from the bacterial host as described in the Methods section and in the Supplementary Information. Analysis of the purified fractions by SDS-PAGE demonstrated that only minor contaminants were present in these fractions (see Supplementary Fig. S2). Immunoblotting of recombinant LeEIN2^462–1316^ using an anti-polyhistidine antibody suggests that these minor contaminating bands correspond to degradation products of the target protein (see Supplementary Fig. S2), rather than impurities from proteins of the bacterial host. Purified recombinant PHOSPHOENOLPYRUVATE-CABOXYLASE (PEPC) expressed and isolated from the same bacterial host strain showed no such contaminating bands when analyzed on the same immunoblot (see Supplementary Fig. S2), supporting the conclusion that the additional bands observed in the LeEIN2^462–1316^ sample reflect degradation products of the recombinant tomato EIN2 protein fragment. Calculation of protein concentration in the purified fractions revealed that about 1 mg of the C-terminal part of LeEIN2 per liter of cells was obtained by this protocol which is comparable in yield with recombinant production of the *Arabidopsis* EIN2 C-terminal domain in *E. coli*^10^.

Recombinant tomato receptor protein LeETR1 required for *in vitro* analysis of complex formation with EIN2 was detergent solubilized and purified to homogeneity according to previous studies^32^ (see Supplementary Fig. S2), in which functionality of the purified tomato receptor protein was also demonstrated^32^.

**Figure 1:**
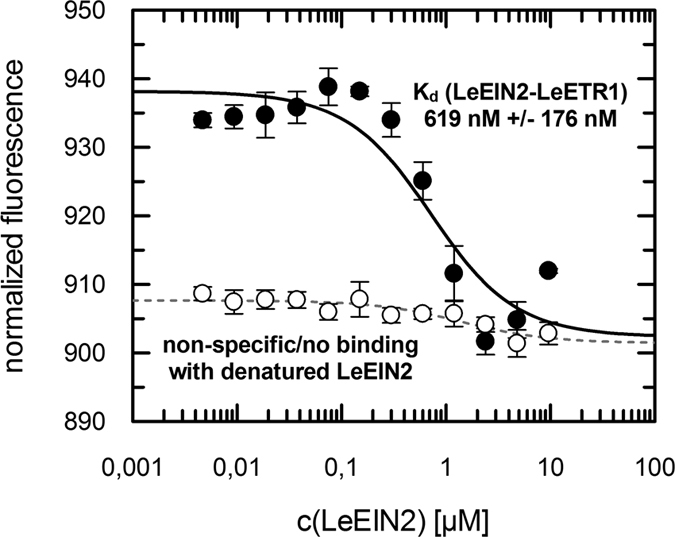
Interaction studies of tomato ETR1 and EIN2 via MST. Determination of *K*_d_ value of LeEIN2-LeETR1 complex formation based on MST-data is illustrated. Normalized fluorescence as MST signal is plotted against increasing LeEIN2^462–1316^ (●) concentrations as described. Binding curve was calculated by a model assuming one binding site per binding partner and resulted in a *K*_d_ = 619 nM +/− 176 nM. As negative control thermally and chemically denaturated LeEIN2^462–1316^ (○) was used. The MST signal indicates no interaction with the purified LeETR1 receptor. All data represent the mean +/− standard deviation.

Protein-protein interaction of LeEIN2 and LeETR1 was studied by microscale thermophoresis (MST). Thermophoresis, the motion of molecules in a temperature gradient, strongly depends on the hydration-shell, the charge and the size of the moving molecules. Typically, at least one of these features changes when a protein-protein interaction occurs. Thus, MST represents a highly sensitive and reliable tool to study protein-protein interactions. By recording the motion of a fluorescence-labeled molecule in the presence of increasing concentrations of a putative binding partner, the dissociation constant (*K*_d_) reflecting the affinity of the interaction and the stability of the complex can be calculated^33^,^34^,^35^. Recombinant LeETR1 was labeled with the fluorescent dye Alexa Fluor 488 and 0.4 μM of labeled LeETR1 was mixed with increasing concentrations of LeEIN2^462–1316^ until saturation was reached (9.6 μM as final concentration). Binding of receptor and EIN2 was observed as a clear and strong response in the MST signal. Analysis of MST signals at different LeEIN2^462–1316^ concentrations revealed that the tomato receptor forms a tight complex with the recombinant C-terminal domain of tomato EIN2. Tight binding of both ethylene pathway proteins is indicated by the low *K*_d_ of 619 nM that is obtained when MST data were fitted according to a cooperative binding model (Fig. 1). A similar high affinity binding constant (651 nM) was found for the interaction of both ethylene proteins when LeEIN2^462–1316^ labeled with Alexa Fluor 488 was titrated with non-labeled LeETR1 (see Supplementary Fig. S3). These controls verify that the fluorescent probe does not affect protein integrity or protein interaction. When thermally and chemically denatured EIN2 was used as negative control, the MST response was non-saturable and reduced below the signal-to-noise ratio indicative of non-specific protein-protein interactions (see Fig. 1). These controls and previous interaction studies with the corresponding *Arabidopsis* proteins *in planta* and *in vitro*^10^,^11^,^26^ underline that EIN2 and ETR1 interact in a selective and specific manner in both plant species.

### NOP-1 inhibits formation of tomato EIN2-ETR1 signaling complex

**Figure 2:**
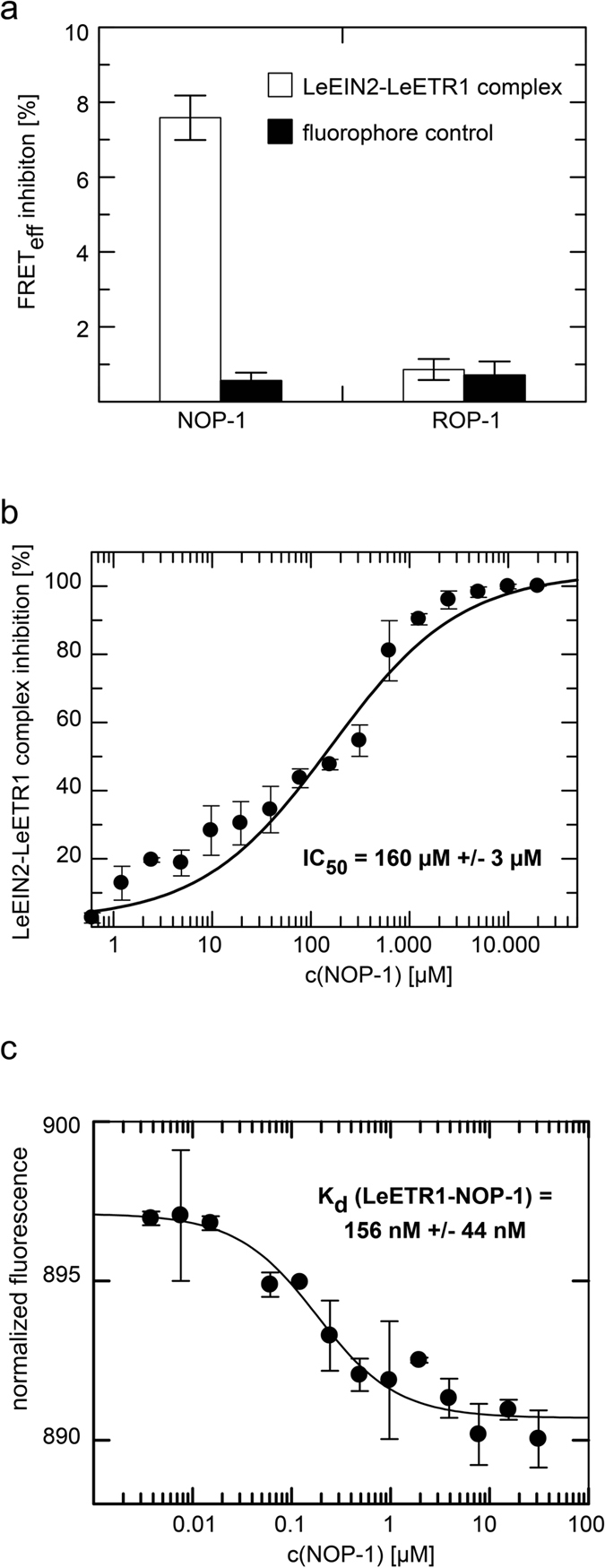
NOP-1 efficiently inhibits LeEIN2-LeETR1 complex formation. (**a**) FRET-based assay reveals that 100 μM NOP-1 leads to a reduced FRET from LeETR1-Alexa Fluor 488 to LeEIN2-Alexa Fluor 568, but not 100 μM of control peptide ROP-1. Neither of both peptides affected fluorescence of the free, unlabeled dyes, reflecting the specific inhibition of complex formation. (**b**) Calculation of *IC*_50_ value for NOP-1. Data were obtained in the FRET-assay in presence of increasing concentrations of NOP-1 peptide. An *IC*_50_ = 160 μM +/− 3 μM was calculated. (**c**) Determination of *K*_d_ value of NOP-1 binding to LeETR1 based on MST is illustrated. MST-signal is plotted against the NOP-1 concentration. A *K*_d_ value of 156 nM +/− 44 nM was calculated. All data (**a**,**b**,**c**) represent the mean +/− standard deviation.

Previous studies from our group indicate that the small octapeptide NOP-1 (LKRYKRRL-NH_2_) derived from the conserved NLS-motif in the C-terminus of EIN2 from *Arabidopsis* is a potent inhibitor of ethylene responses in living plants^26^. Inhibition of ethylene responses by the small peptide is related to a reduced or abolished EIN2-ETR1 interaction in *Arabidopsis*^26^. Hence, we next analyzed the effect of NOP-1 on the tomato EIN2-ETR1 complex formation. For this purpose, we made use of a fluorescence resonance energy transfer (FRET)-based *in vitro* assay, originally established for the respective *Arabidopsis* signaling proteins^26^. Recombinant ethylene proteins LeETR1 and LeEIN2^462–1316^ were labeled with fluorescent Alexa dyes^36^ as described in the Methods section. LeETR1 was labeled with Alexa Fluor 488, while LeEIN2^462–1316^ was labeled with Alexa Fluor 568. As the emission spectrum of the donor fluorophore partially overlaps with the excitation spectrum of the acceptor fluorophore, the dyes form a FRET pair and energy transfer from the donor to the acceptor fluorophore is possible when both probes get in close proximity^37^. Both proteins were mixed at a final concentration of 3 μM, corresponding to a concentration 5-fold the *K*_d_ of the tomato EIN2-ETR1 complex (see above and Fig. 1). Formation of the complex is monitored by FRET from the donor molecule Alexa Fluor 488 to the acceptor molecule Alexa Fluor 568. Processes or molecules that prevent complex formation result in a reduced FRET from the donor to the acceptor, which is detected as reduced acceptor fluorescence at the emission maximum^26^. Measurements in the presence of 100 μM NOP-1 (Fig. 2a) show a pronounced inhibition in FRET efficiency (about 9% of the value observed in the absence of NOP-1) similar to the effect of the peptide on the *Arabidopsis* EIN2-ETR1 complex. When an octapeptide of random sequence (EFLYMSVN-NH_2_) termed ROP-1 (random octapeptide-1) was applied in the FRET assay, no effect on tomato EIN2-ETR1 complex formation was observed. As fluorescence of the dyes on their own was not affected by the peptides (Fig. 2a), reduced FRET efficiency observed in the presence of NOP-1 can be attributed to dissociation of tomato EIN2-ETR1 complex. To further characterize the inhibitory effect of NOP-1 on this complex, the FRET-assay was carried out at increasing NOP-1 concentrations. When reduced FRET efficiencies were plotted versus the different NOP-1 concentrations, a half maximal inhibitory concentration (*IC*_50_) of 160 μM was determined for the NOP-1 peptide (Fig. 2b). This value corresponds to the *IC*_50_ obtained for the inhibitory effect of NOP-1 on the interaction of the related proteins from *Arabidopsis* (93 μM)^26^.

To substantiate the inhibitory role of NOP-1 in LeEIN2-LeETR1 complex formation, NOP-1 binding to LeETR1 was studied by microscale thermophoresis (MST) as described in the Methods section. The binding curve reflecting the interaction of NOP-1 with the LeETR1 receptor (Fig. 2c) is characterized by a *K*_d_ of 156 nM +/− 44 nM indicating a highly specific interaction of NOP-1 with the tomato ethylene receptor. The *K*_d_ for the LeETR1-NOP1 interaction compares to the affinities of the EIN2-ETR1 interaction in *Arabidopsis*^10^,^11^ and tomato (this work) emphasizing that the NOP-1 peptide can efficiently compete for ETR1 binding and disrupt EIN2-ETR1 complex formation.

### NOP-1 delays tomato fruit ripening by inhibiting ethylene perception

**Figure 3:**
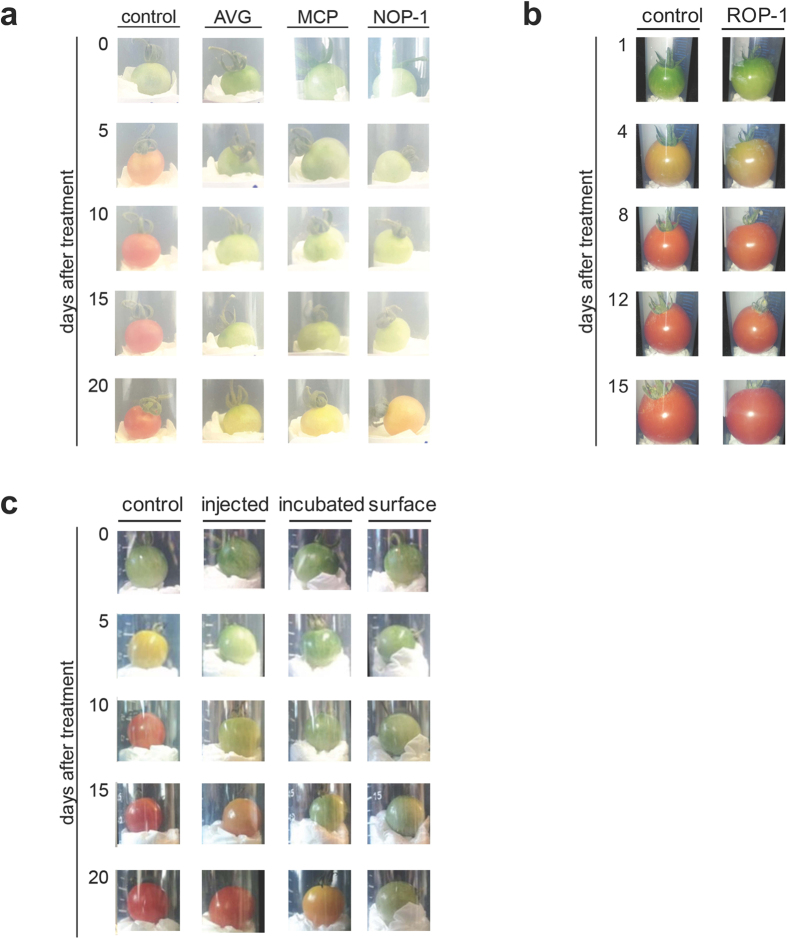
Inhibition of tomato fruit ripening process by treatment with different substances. Illustrated are representative pictures of the tomato fruit ripening study. (**a**) Immature tomato fruits were surface-treated with 200 μM NOP-1, 200 μM AVG and 200 μM MCP, respectively. A control tomato fruit population remained untreated. All NOP-1 treatments led to a delay in fruit ripening of at least 15 days, representing the high inhibitory potential of the synthetic peptide reflecting the EIN2-NLS motif to affect fruit ripening as ethylene response. (**b**) A related delay in ripening was not observed when fruits were treated with buffer only or treated with random sequence control peptide ROP-1(EFLYMSVN) (adopted from Ref. 26). (**c**) Green, immature tomato fruits were surface-treated, incubated or injected with 200 μM of NOP-1 as described. Control tomato fruits remained untreated. All applications of NOP-1 led to a delay in fruit ripening of at least 5 days. Delay in maturation process is most evident for fruit surface application.

To further investigate the potential of NOP-1 for agricultural applications, we tested the effect of the NOP-1 peptide on tomato fruit ripening in comparison to other known inhibitors of ethylene responses. To this end we tested 1-methylcyclopropane (MCP) and aminoethoxyvinylglycine (AVG). MCP is a known inhibitor of ethylene signaling blocking ethylene binding at the receptor proteins, whereas AVG blocks ethylene production in the plant by competitive inhibition of ACC synthases^20^,^38^. To monitor inhibition of the fruit ripening process, we treated green, immature tomato fruits with 200 μM NOP-1, MCP or AVG, respectively and documented the fruit ripening process for 20 days. Figure 3 compares the effect of the different inhibitor treatments on tomato fruits to controls lacking the inhibitory treatment that contain either buffer alone or a random sequence control peptide (ROP-1). A delay of 10–15 days in maturation was observed for all tested inhibitors and for all applications of NOP-1 (injection, incubation, surface application) tested in the experiments. Like buffer treatment ROP-1 had no effect on the ripening process (see Fig. 3b). The bioassays underline the high inhibitory potential of NOP-1 on the ripening process compared to the well-known and efficient inhibitors of ethylene responses. The fact that surface application (see Fig. 3a,c) is sufficient to delay ripening implies that the NOP-1 peptide can easily penetrate the surface tissue of the tomatoes and is taken up by the fruits.

**Figure 4:**
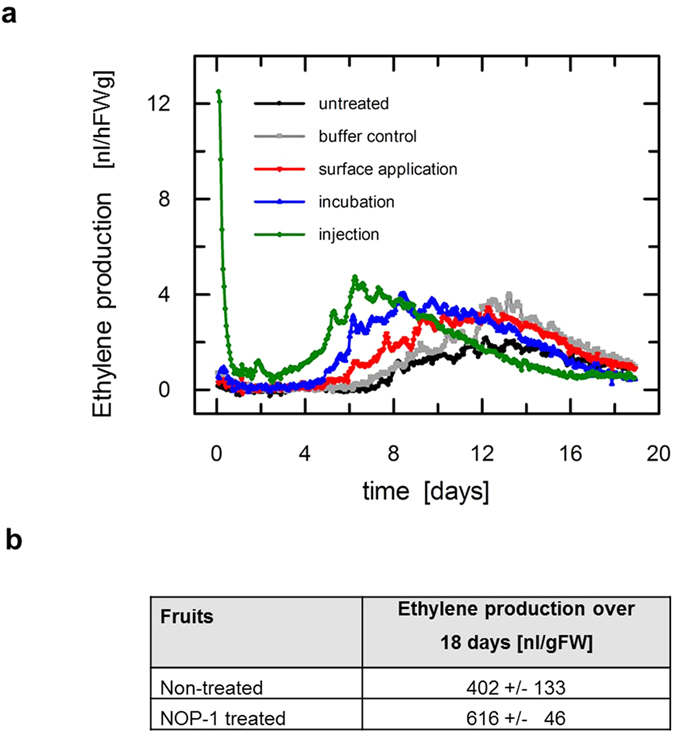
Ethylene production of climacteric tomato fruits previously treated with NOP-1 at different applications. (**a**) Real-time monitoring of ethylene released by fruits treated with 200 μM NOP-1 (see insert for application form of the peptide) compared to untreated fruits and buffer control. Measurements were stopped when fruits showed a fully ripe phenotype. The initial burst in ethylene production within the first hours in fruits injected with the peptide is attributed to wounding. No reduction in ethylene production is evident in any of the different NOP-1 applications compared to buffer control or untreated fruits highlighting that ripening-delay caused by NOP-1 is not associated with lower levels in ethylene biosynthesis. The overall pattern of ethylene release is similar for all treatments, aside from the initial ethylene burst in the injected fruits and a peak shift in fruits incubated or injected with the peptide. (**b**) Average numbers of total ethylene release over 18 days in non-treated fruits (untreated and buffer control) compared to fruits treated with NOP-1 at different applications (surface-application, incubation and injection). In these calculations total ethylene release in injected fruits was corrected for the initial ethylene burst.

To further analyze the mode of inhibition by NOP-1, we asked whether the effect of NOP-1 is based only on disruption of ethylene perception or might also result from inhibition of ethylene biosynthesis in the fruit. To address this question we measured production of endogenous ethylene of climacteric tomato fruits treated with NOP-1 in various application forms (for details see methods). Ethylene production of fruits was measured using a laser-based ethylene detector. To minimize the effects of individual variation we used 6 fruits per cuvette per treatment. As illustrated in Fig. 4a, aside from an ethylene burst detected in the injected fruits, the release of ethylene from NOP-1 treated tomato fruits show a similar curve progression and do not significantly differ from controls (untreated fruits and tomatoes placed in buffer lacking the peptide)–no matter on the application method used (surface treatment, injection of or incubation in peptide solution). While ethylene production in surface-treated and control fruits peaks at the same time (~day 12), fruits injected or incubated with NOP-1 seem to peak earlier (day 6–8) which might be related to an abiotic stress-response triggered by mechanical wounding (injection) or submergence (incubation). Both processes are well-known for a related increase in ethylene production^39^,^40^,^41^. Nonetheless, the full significance of the observed shift is not clear yet and needs a further, more detailed investigation.

Altogether, on average the different NOP-1 treatments show a modest increase rather than an inhibition of ethylene production (see Fig. 4b) ruling out any inhibitory effect of the peptide on ethylene biosynthesis (e.g. ACC synthase or ACC oxidase) and a related delay in ripening by lower levels of ethylene.

## Discussion

We found that NOP-1, a small basic octapeptide derived from the highly conserved NLS sequence in the C-terminal part of ethylene signaling protein EIN2, is a potent inhibitor of ethylene responses in climacteric tomato fruit. The inhibitory mode of NOP-1 is based on disruption of the EIN2-ETR1 complex which forms a conserved motif of ethylene signaling in *Arabidopsis* and tomato. Similar high affinity dissociation constants were determined for the EIN2-ETR1 interaction in tomato (see Fig. 1) and in *Arabidopsis*^10^,^11^. Taking into account that both plants are evolutionary separated by 150 mya^42^,^43^, complex formation between EIN2 and ETR1 is likely to reflect a conserved mechanism in ethylene signal transduction in a wide range of plant species. Our data suggest that the highly conserved NLS sequence provides the binding site in EIN2 for interaction with the receptors in both, *Arabidopsis*^26^ and tomato (this work).

Previous studies on *Arabidopsis* ethylene signaling proteins ETR1 and EIN2 demonstrated that the basic octapeptide NOP-1 is able to disrupt interaction of EIN2 and ETR1^26^. Studies on the related tomato proteins in this work emphasize that NOP-1 follows the same inhibitory mechanism also in other plants. The NLS-based peptide leads to specific disruption of the tomato EIN2-ETR1 complex (see Fig. 2a). The *IC*_50_ of 160 μM determined for inhibition of the tomato EIN2-ETR1 interaction by NOP-1 (see Fig. 2b) corresponds to *IC*_50_ values obtained for other protein-protein interaction inhibitors in previous studies^44^. Similar *IC*_50_ values for inhibition of the EIN2-ETR1 interaction in *Arabidopsis* (93 μM)^26^ and tomato (160 μM, this work) suggest that NOP-1 is a potential inhibitor of ethylene responses in a wide range of plants.

NOP-1 binding to LeETR1 is characterized by a *K*_d_ value of ~150 nM (see Fig. 2c). This value compares to binding constants of previously described inhibitors of protein–protein interactions^44^ underlining the high potential of NOP-1 to inhibit EIN2-ETR1 complex formation. The high affinity binding constant of ETR1 for NOP-1 similar to that for EIN2 suggests that NOP-1 and EIN2 compete for the same binding site at the receptor. Furthermore, a competitive inhibitory mechanism is in accordance with the delayed or reduced ethylene responses that were observed upon NOP-1 treatment in living plants (Ref. 26 and this work).

In order to evaluate the inhibitory potential of the NLS-based peptide, we compared the result of NOP-1 and the well-known inhibitors of ethylene perception and biosynthesis, MCP and AVP, on fruit ripening. We observed a similar delay in tomato fruit ripening by NOP-1, AVG and MCP suggesting that NOP-1 has high potential to delay ripening. As NOP-1 in our studies was applied on the fruit surface, the peptide seems to penetrate the exocarp and is taken up by the fruits, similar to established ripening inhibitors. NOP-1 uptake might be catalyzed by the Oligopeptide Transporter (OPT) Family^45^,^46^,^47^ enabling transport of tetra-, penta-, hepta and octapetides across cell membranes or due to the inherent characteristics of the peptide, i.e. short length (upper limit 30–35 residues), water-solubility, partly hydrophobic and/or polybasic composition and positive net charge at physiological pH^48^,^49^. Based on the observed direct uptake of the compound from the fruit surface, alternative application forms of NOP-1 such as repetitive spraying or delayed release may provide an even more efficient ripening control. Injection of NOP-1 into the fruits, which was tested in our initial experiments, produced a less pronounced delay in ripening probably due to the related ethylene burst produced by the mechanical injury of the fruits (see Fig. 4a). Alternatively, uptake of NOP-1 from the fruit surface is gradual and external NOP-1 more stable than the internalized peptide which would also account for the less efficient ripening delay observed in the injection approach.

Due to its short length, the NOP-1 peptide probably lacks any tertiary structure. Thereby, the peptide is stable for several days at room temperature under normal conditions and is not prone to thermal denaturation as most proteins are. This postulated long-term stability of NOP-1 was experimentally demonstrated by LC/MS (see Supplementary Fig. S4). In the same way, reactions that could damage the peptide, such as oxidation, require high or low pH and are very slow under the pH condition of biological systems. The only way to damage the peptide is covalent modification or breaking of peptide bonds. Thus, N- and/or C-terminal capping of the peptide or use of d-amino acids might provide longer lasting effects on ripening delay. Of course, potential toxicity or immunogenicity is a critical concern on the use of a peptide for agricultural or horticultural application. Several techniques to measure cytotoxicity of a peptide *in vitro* such as MTT assay, LDH leakage assay or ATP-based assay have been applied for various potential therapeutics^50^. In addition, several *in silico* sequence-based tools are available to predict toxicity or immunogenicity. Sequence analysis of NOP-1 by ToxinPred^51^ and Allergenic Protein Sequence Search in the AllegenOnline.org database revealed no particular risk that is associated with the NOP-1 peptide. Thus, to the best of our knowledge we are not aware on any toxicity or allergenic effects of such a short basic peptide as NOP-1 on any organism. Today, production of NOP-1 or related peptides is presumably more costly than chemical synthesis of the inhibitors MCP or AVG. However, peptide production costs have been substantially reduced in recent years due to recombinant technologies and improved solid or liquid phase synthesis. Further cost reduction is expected making commercial application of peptides feasible also for agricultural or horticultural markets.

Taken together, our study demonstrates that the small basic peptide NOP-1 derived from the natural NLS-sequence of the ethylene regulator protein EIN2 is a potent inhibitor of the maturation process in tomato fruits. Other climacteric fruits and vegetable might respond in a similar way as the mode of action by which the peptide interferes with ethylene signaling is conserved in evolutionary distinct plants such as *Arabidopsis* and tomato. The inhibitory effect of NOP-1 is related to destabilization or even inhibition of EIN2-ETR1 complex formation, which seems to be a conserved module in ethylene signaling.

## Methods

### Cloning of the C-terminal domain of tomato ethylene regulator LeEIN2^462–1316^

For vector construction, expression vector pET-15b (Novagene) was used as a backbone. First, 6×-His-tag of pET-15b was upgraded to a 10×-His-tag by QuickChange II Site-Directed Mutagenesis Kit (Agilent Technologies) following the manufacturer’s instructions. Mutagenic primer sequences were 5′-TATACCATGGGCAGCAGCCATCATCATCATCATCATCATCATCATCACAGCAGCGGCCTG-3′ for the forward primer and 5′-CAGGCCGCTGCTGTGATGATGATGATGATGATGATGATGATGGCTGCTGCCCATGGTATA-3′ for the reverse primer. Resulting plasmid pET-15b_10×His_ was used as a backbone for insertion of additional recognition sites for restriction enzymes *Nhe*I, *Kpn*I and *Xma*I. QuickChange II -Kit was again used for insertion. Mutagenic primer sequences were 5′-AGCTTTCATATGGCTAGCGGTACCGCACCCGGGCTCGAGGATC-3′ for the forward primer and 5′-GATCCTCGAGCCCGGGTGCGGTACCGCTAGCCATATGAAAGCT-3′ for the reverse primer. The obtained plasmid was sequenced to confirm insertions and to exclude additional mutations. The final plasmid containing all insertions was named pET-15b_plus_.

cDNA encoding for full-length LeEIN2 (UniProt ID: Q6Q2C1) was commercially purchased at GenScript USA Inc. The cDNA sequence was ordered according to the published sequence (NCBI ID: NM_001247589.1)^29^, 5′-flanked by a recognition site for restriction enzyme *Nhe*I and 3′-flanked by a recognition site for *Xho*I. Synthetic DNA was digested with *Nhe*I and *Xho*I and ligated into expression vector pET-15b_plus_, previously linearized by the same enzymes. Resulting plasmid pET-15b_plus__LeEIN2 was used as a backbone to delete the N-terminal membrane domain (amino acid aa 1-461) of LeEIN2. To this end, the Phusion Site-Directed Mutagenesis Kit (Thermo Scientific) was used following the manufacturer’s instructions. Mutagenic primer sequences were 5′-[Phos]CTGAAATCTGCAAGTTCC-3′ for the forward primer and 5′-[Phos]CATGCTAGCCATATGG-3′ for the reverse primer. The resulting plasmid was sequenced for successful deletion and termed pET-15b_plus__LeEIN2^462–1316^.

### Expression and purification of recombinant proteins

The expression vector containing the soluble C-terminal part of tomato EIN2 (pET-15b_plus__LeEIN2^462–1316^) was transformed into *E. coli* strain BL21(DE3). Plasmid pRARE (Novagene) was co-transformed to provide essential tRNAs encoded by rarely used codons in *E. coli* and cells were grown in 2YT medium (1.6% (w/v) peptone, 1% (w/v) yeast extract and 0.5% (w/v) NaCl) at 30 °C. Protein expression was induced by addition of 0.5 mM isopropyl-β-d-1-thiogalactopyranoside (IPTG) when *OD*_600_ = 0.6 − 0.8 was reached. Cells were harvested 4–5 h after induction by centrifugation. Resulting cell pellet was resuspended in 50 mM Tris-HCl pH 7.6, 300 mM NaCl, 6% (v/v) glycerol and 5 mM dithiothreitol. DNAseI (10 μg/mL) and 1 × EDTA-free cOmplete Protease Inhibitor Cocktail from Roche were added to the cell suspension prior to cells were disrupted by passing through a pre-cooled French pressure cell at 12000 psi (1 psi = 6.9 kPa). The supernatant of the cell lysate after ultra-centrifugation (100,000 × g, 2 h, 4 °C) was loaded onto a 5 mL HisTrap FF column at 4 °C connected to an ÄKTAprime plus (both GE Healthcare Life Sciences), followed by an washing step with adenosine triphosphate (ATP) (resuspension buffer plus 50 mM KCl, 20 mM MgCl_2_ and 10 mM ATP). After a second wash step with 50 mM imidazole, LeEIN2 was eluted with 500 mM imidazole. The purified protein was concentrated to 2.5 mL and buffer was exchanged by a desalting step on a PD-10 column (GE Healthcare Life Sciences). Receptor protein LeETR1 from tomato was heterologously expressed in *E. coli* and purified from the bacterial host according to Ref. 32. PEPC from *Flaveria trinervia* was expressed and purified according to Ref. 52. Recombinant proteins were quantified using the BCA assay (Life technologies). Expression and purification of tomato proteins were analyzed by SDS-PAGE (sodium dodecyl sulfate-polyacrylamide gel electrophoresis) on 10% SDS-gels^53^ and stained by Coomassie^54^. For immuno-detection by western blot^55^ or dot blot^56^ directly conjugated Anti-His-HRP monoclonal antibody (Milteny Biotech) was used.

### Quantitative interaction studies by MST

Analysis of LeETR1-LeEIN2 complex formation, NOP-1 binding to LeETR1 and determination of the respective dissociation constants *K*_d_ was performed by MST^33^,^34^,^35^. For protein-protein interaction studies LeETR1 was labeled with Alexa Fluor 488 succinimidyl-ester (Life Technologies) in a buffer containing 50 mM potassium phosphate pH 8.0, 300 mM NaCl and 0.015% (w/v) Fos-Choline-16 according to the manufacturer’s protocol. Labeled LeETR1 was transferred in 50 mM Tris-HCl pH 8.0, 300 mM NaCl, 0.015% (w/v) Fos-Choline-16 and purified LeEIN2^462–1316^ was serially diluted in the same buffer in a 1:1 ratio for 12 times, resulting in 9.6 μM as highest concentration and 4.69 nM as lowest LeEIN2^462–1316^ concentration. These different concentrations of LeEIN2^462–1316^ were subsequently mixed with 0.4 μM of labeled LeETR1 -Alexa Fluor 488 and samples were transferred into standard glass capillaries for MST. Measurements were carried out using a Monolith NT.115 (NanoTemper Technologies) at 80% MST power in independent triplicates. Thermally and chemically denatured LeEIN2^462–1316^ was taken for control measurements to demonstrate selectivity and specificity of binding. To this end, LeEIN2^462–1316^ was first heated at 95 °C for 5 minutes. Then, the thermally denatured protein was diluted in the buffer previously described. The strong denaturing detergent sodium dodecyl sulfate (SDS) at a concentration of 4% (v/v) and 40 mM DTT were added to each dilution. Finally, samples were mixed with the labeled LeETR1. For titration the denatured protein was used at the same concentrations in the MST experiments as the native LeEIN2^462–1316^. Again measurements were done in triplicates.

For quantification of NOP-1 binding to tomato receptor protein, recombinant LeETR1 was labeled with Alexa Fluor 488-Maleimide (Life Technologies) in a buffer containing 50 mM potassium phosphate pH 8.0, 300 mM NaCl, 0.015% (w/v) Fos-Choline-16 following the manufacturer’s protocol. NOP-1 was resuspended in a buffer containing 50 mM Tris-HCl pH 8.0, 300 mM NaCl, 0.015% (w/v) Fos-Choline-16 and was serially diluted as described above, resulting in 3.8 nM as lowest concentration and 31 μM as highest concentration of the peptide ligand. Diluted NOP-1 was subsequently mixed with labeled LeETR1 in a 1:1 ratio, resulting in 50 nM final concentration of the labeled protein. Protein-peptide mixtures were transferred into glass capillaries and MST was measured with 80% MST power. MST signals were fitted against the concentration of the labeled binding partner using the program GraFit (Erithacus Software). The dissociation constant *K*_d_ was calculated according to a model assuming a 1:1 stoichiometry per binding partner.

### Quantitative analysis of inhibitory peptides on ETR1-EIN2 complex by FRET

For the FRET assay, recombinant LeETR1 was labeled with Alexa Fluor 488 succinimidyl-ester and LeEIN2^462–1316^ was labeled with Alexa Fluor 568 succinimidyl-ester (both Life Technologies) in a buffer containing 50 mM potassium phosphate pH 8.0, 300 mM NaCl and 5% (v/v) glycerol following manufacturer’s protocol (MolecularProbes Protein Labeling Kit). Labeled proteins were transferred in FRET buffer containing 50 mM Tris-HCl pH 8.0, 300 mM NaCl, 0.05% (v/v) Tween 20, 5% (v/v) glycerol and mixed at a final concentration of 3.0 μM each. For tests on the effect of synthetic peptides on complex formation, proteins were pre-incubated in the presence of these peptides before the measurements. The FRET-based binding assay was carried out in 384-well microplates using an Infinite M200 Pro microplate reader (Tecan) at 455 nm excitation wavelength. Emission spectra were detected between 565 nm to 645 nm. FRET efficiencies were calculated from the maximal emission in the absence and in the presence of the supplied peptides and the half maximal inhibitor concentration *IC*_50_ was calculated as previously described^26^. Peptides NOP-1 and ROP-1 were synthesized at the Molecular Proteomics Laboratory of the BMFZ Düsseldorf with >90% purity level, N-terminal free amine and C-terminal blocked amidation.

### Inhibitor-treatment of tomato fruits

Aminoethoxyvinyl glycine (AVG) was purchased from Sigma-Aldrich, St Louis, MO (purity ~95%). SmartFresh^SM^ (AgroFresh, Spring House, PA) was used as sugar-based powder formulation of 1-MCP. Concentration of 1-MCP was calculated according to the percentage of active ingredient (0.14%) and release from the SmartFresh^SM^ powder. For comparison concentration of all inhibitors are expressed in terms of molarity. For treatment of tomato fruits with NOP-1, AVG and MCP, inhibitors were dissolved in the FRET buffer described in the previous section to a final concentration of 200 μM. Green tomato fruits (*Solanum pimpinellifolium*) from the same estimated developmental stage were harvested from the panicle and water-cleaned. 300 μL of the inhibitor solution was applied onto the tomato surface with a brush and the fruits were air-dried for 30 min. As an alternative application, tomato fruits were plunged into 2 mL of the inhibitor solution and incubated for 30 min in this solution or 200 μL of the peptide-containing solution were carefully injected into the tomato tissue by a hypodermic syringe. Three individual tomato fruits were treated for the different applications of the peptide in order to document the ripening process. After inhibitor-treatment tomato fruits were transferred to a vial closed with aluminum foil and stored in the dark at room temperature. Maturation process was documented every second day for at least 20 days. For measurement of ethylene production, green tomato fruits were treated as described above.

### Real-time monitoring of ethylene production

Ethylene production was continuously monitored over 19 days with a laser-based ethylene detector (ETD-300; Sensor Sense BV; Nijmegen, Netherlands). A detailed description of the system has been given elsewhere^57^. Briefly, the detector consists of a laser emitting radiation in the 10 micrometers infrared wavelength region and a photoacoustic cell, in which ethylene is detected due to its strong fingerprint-like absorption pattern in this region. Inside the photoacoustic cell ethylene can absorb the laser radiation and converts it into kinetic energy by energy exchange processes, resulting in local heating of the sample. By switching on and off the light, rapid heating/cooling can occur, giving rise to a periodical pressure change, i.e. sound wave. The amplitude of these waves is directly proportional to the concentration of ethylene in the photoacoustic cell and can be detected very accurately with sensitive miniature microphones.

Six tomato fruits for each treatment, including an untreated group (buffer control, CT) were placed into glass cuvettes (150 ml volume) and connected to a gas flow through system (VC-6, Sensor Sense BV; Nijmegen, Netherlands) under a continuous flow routine as described in Ref. 58. The VC-2 ensured that the samples were continuously flushed with air at a constant flow of 2 l/h and allowed automated sampling of ethylene from the headspace of six cuvettes. Each cuvette was alternatively connected to the ETD-300 and ethylene emitted in the headspace was measured for 12 min. During the measurements the cuvettes were kept in an environmental chamber under dark and at constant temperature of 21 °C (Sanyo MLR-350 H; Light source: Phillips, TL-D-36W/33-640SLV). A scrubber with KOH (moist pellets) and CaCl_2_ (granules) was used before the ETD-300 to reduce the CO_2_ concentration to less than 1 ppm and the water content in the gas flow, respectively. The ethylene production was related to the emission rate by multiplying the measured value with the flow rate (expressed in nanoliters per hour, nl/h) and further normalized with the fresh weight (expressed in grams, gFW); the end result being nanoliters per hour per gram of fresh weight, nl/h∙gFW.

## Additional Information

**How to cite this article**: Bisson, M. M. A. *et al.* Peptides interfering with protein-protein interactions in the ethylene signaling pathway delay tomato fruit ripening. *Sci. Rep.* **6**, 30634; doi: 10.1038/srep30634 (2016).

## Supplementary Material

Supplementary Information
