# Supplementary material for: Peptides interfering with protein-protein interactions in the ethylene signaling pathway delay tomato fruit ripening
Source: Sci Rep. 2016 Aug 1;6:30634. doi: 10.1038/srep30634 (PMC4967898; doi:10.1038/srep30634)
Supplement: Supplementary Information [file srep30634-s1.pdf]

Peptides interfering with protein-protein interactions in the ethylene signaling pathway delay tomato fruit ripening

Melanie M.A. Bisson[1, #], Mareike Kessenbrock[1, #], Lena Müller[1], Alexander Hofmann[1], Florian Schmitz[1], Simona M. Cristescu[2] and Georg Groth[1*]

[1] Biochemical Plant Physiology, Heinrich-Heine-University Düsseldorf, D-40204 Düsseldorf, Germany

[2] Department of Molecular and Laser Physics, Radboud University, Heyendaalseweg 135, 6525 AJ Nijmegen, The Netherlands

* Corresponding author: georg.groth@hhu.de

[#] These authors contributed equally to this work

Supplementary Figures

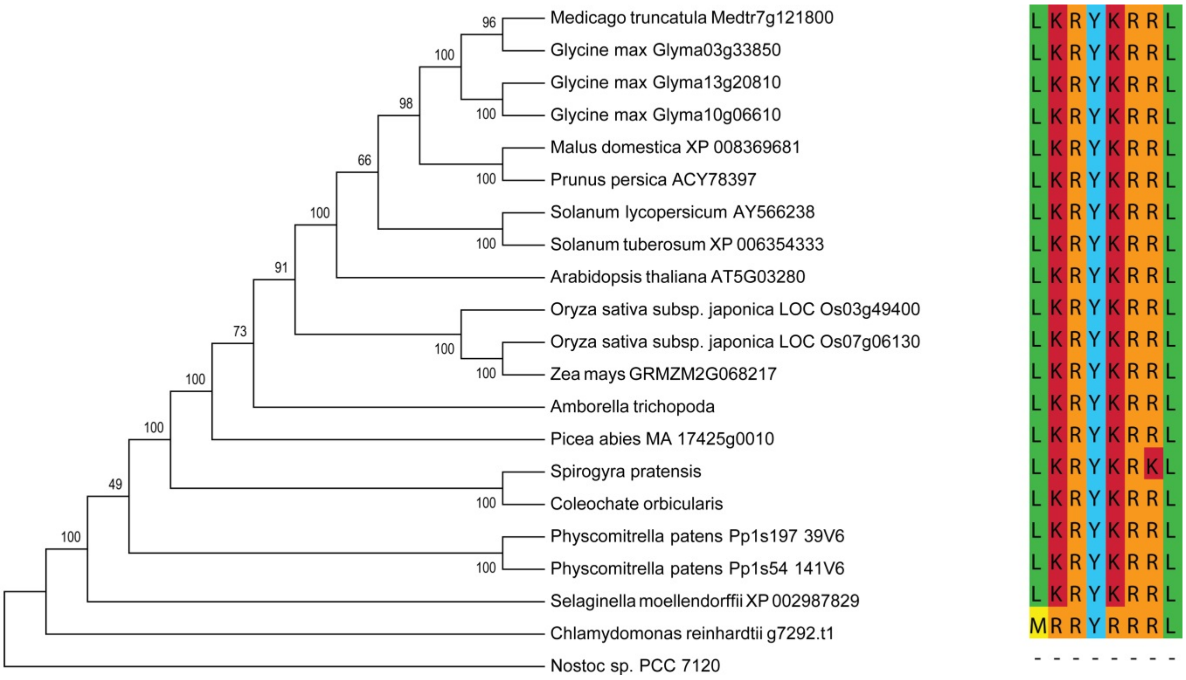

Figure S1: Phylogenetic analysis of EIN2 protein sequences from selected organisms. On the left the phylogenetic analysis of EIN2 protein sequences from selected organisms is depicted. Sequences were retrieved as the best BLASTp hits (best two for *Oryza sativa* subsp. *Japonica*, *Physcomitrella patens* and three for *Glycine max* respectively)[1] at NCBI (http://blast.ncbi.nlm.nih.gov/Blast.cgi) against AtEIN2 (at5g03280). The sequences for *Amborella trichipoda*, *Physcomitrella patens*, *Selaginella moellendorffii* and *Chlamydomonas reinhardti* were acquired as the best BLASTp hits from Phytozome (http://phytozome.jgi.doe.gov). The sequence for *Picea abies* was obtained by BLASTp via Congenie (www.congenie.org) and for *Spirogyra pratensis* (NCBI TSA accession number GBSM01000000) and *Coleochate orbicularis* (NCBI TSA accession number GBSL01000000) as described in ref. 2. Sequences were aligned with the MUSCLE plugin[3] and analyzed in the MEGA6[4] suite. Maximum likelihood analysis was performed with 1000 Bootstrap iterations. Bootstrap values are indicated at the corresponding nodes. The tree was rooted on *Nostoc* sp. PCC 7120. On the right the alignment of the conserved octapeptide is shown corresponding to the protein sequence in the cladogram and colored according to the amino acid side chain.

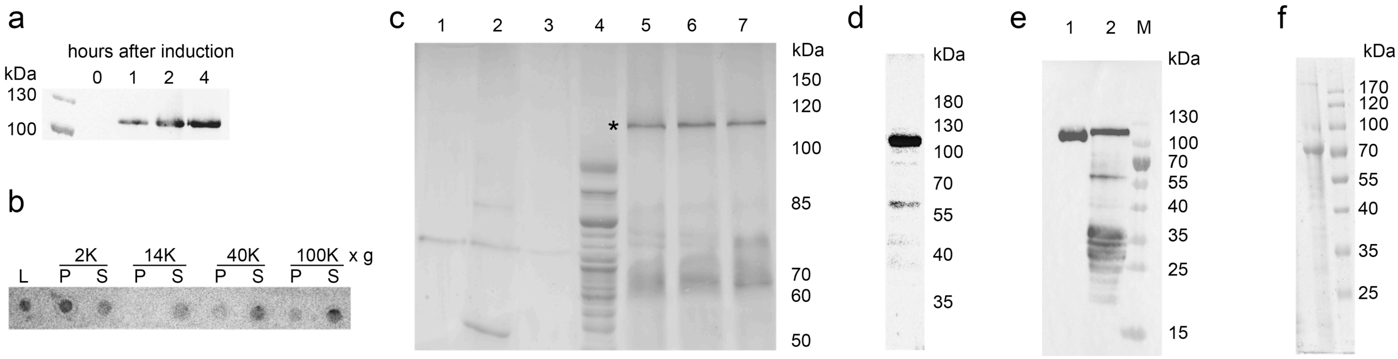

Figure S2: Heterologous expression and purification of the C-terminal domain of ethylene regulator LeEIN2$^{462-1316}$ and tomato ethylene receptor LeETR1. (a) Western Blot analysis with anti-His antibody reveals proper expression of LeEIN2$^{462-1316}$ in *E. coli* after induction with IPTG. (b) Dot-Blot analysis of sequential centrifugation studies of a cell lysate expressing LeEIN2$^{462-1316}$ using anti-His antibody. L = lysate, P = pellet, S = supernatant. Largest amount of recombinant protein was found in the supernatant after centrifugation at 100,000 x g reflecting protein production in soluble form. (c) Coomassie-stained SDS-gel with samples of LeEIN2$^{462-1316}$ purification. 1-3 = ATP-washing step, 4 = 50 mM imidazole-washing step, 5-7 = elution step, * = LeEIN2$^{462-1316}$ protein. Removal of chaperones (1-3) and nonspecifically bound proteins (4) leads to purified protein after elution (5-7). (d) Western Blot analysis with anti-His antibody identifies additional bands in the lower molecular range of a LeEIN2$^{462-1316}$ sample as degradation bands. (e) Identity of degradation bands in LeEIN2$^{462-1316}$ sample (lane 2) is proven by co-blotting of PEPC (lane 1). PEPC, previously expressed and isolated from the same *E. coli* host strain, shows no such degradation bands in a Western Blot analysis with anti-His antibody. (f) Coomassie-stained SDS-gel with a purified sample of tomato ethylene receptor protein LeETR1 reflects high purity of recombinant, detergent-solubilized receptor protein.

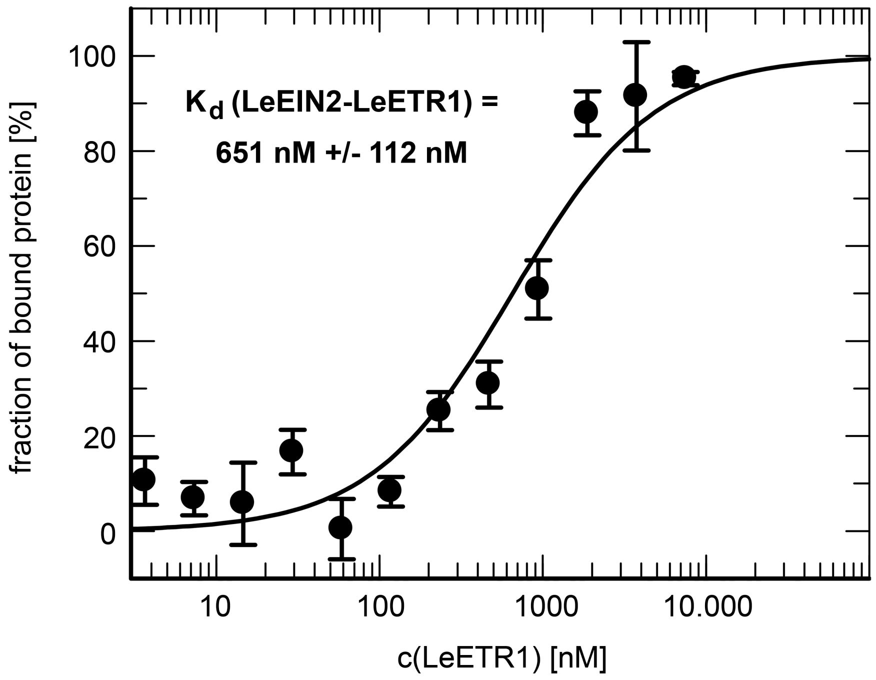

Figure S3: Interaction studies of tomato ETR1 and EIN2. Determination of K_d value of LeEIN2-LeETR1 complex formation based on MST-data is illustrated. LeEIN2$^{462-1316}$ was labelled with Alexa Fluor 488 succinimidyl-ester (Life Technologies) in a buffer containing 50 mM potassium phosphate pH 8.0, 300 mM NaCl and 5 % (v/v) glycerol according to the manufacturer's protocol. Labelled LeEIN2$^{462-1316}$ and LeETR1 were transferred in 50 mM Tris-HCl pH 8.0, 300 mM NaCl, 0.015 % (w/v) Fos-Choline-16 and purified LeETR1 was serially diluted in the same buffer in a 1:1 ratio for 12 times, resulting in 7.5 µM as highest concentration and 3.66 nM as lowest LeETR1concentration. These different concentrations of LeETR1 were subsequently mixed with 0.2 µM of labelled LeEIN2$^{462-1316}$-Alexa Fluor 488 and samples were transferred into standard glass capillaries for MST. Measurements were carried out using a Monolith NT.115 (NanoTemper Technologies) at 60 % MST power. Fraction of bound LeETR1 was fitted against increasing LeETR1 concentrations. Binding curve was calculated by a model assuming one binding site per binding partner and resulted in a K_d = 651 nM +/- 112 nM. Data represent the mean of three independent measurements +/- standard deviation.

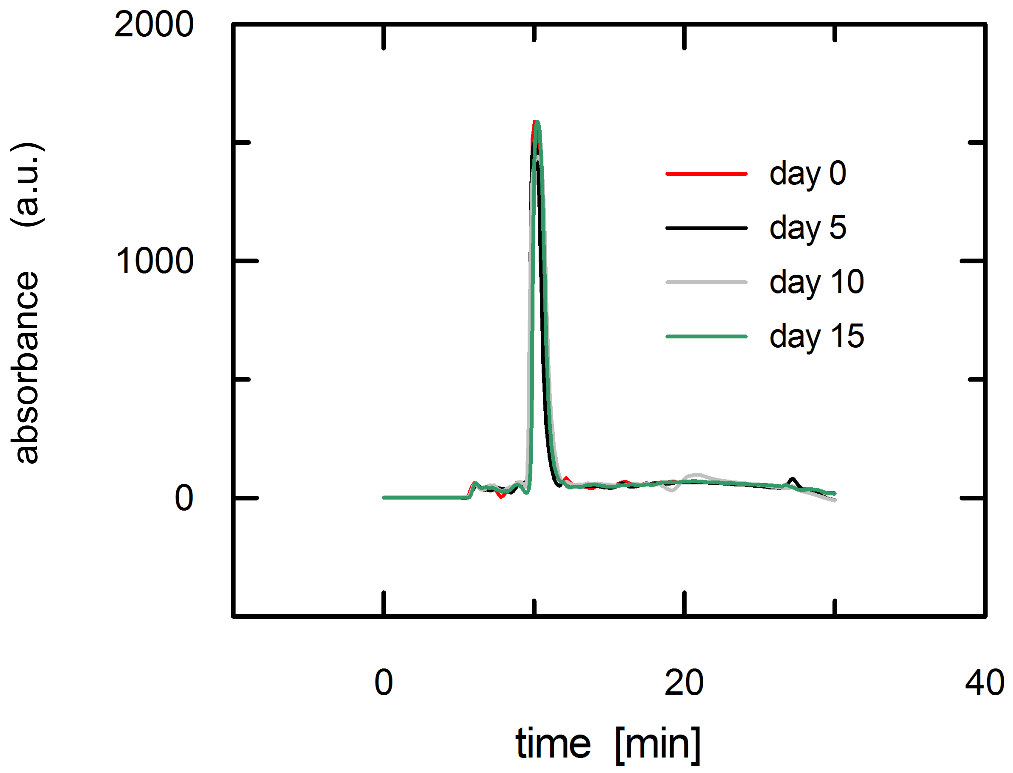

Figure S4: Analysis of NOP-1 stability by liquid chromatography (LC).

Lyophilized NOP-1 (500 µg) was dissolved in 100 µl PBS pH 6.7, incubated for 0 days, 5 days, 10 days and 15 days at room temperatures and then applied to a Supelcosil C18 column (4.6x150 mm). The column was washed with 15 ml H_2O with 0.1% TFA (buffer A). Elution was performed with a linear gradient to 100% buffer B (100% acetonitrile with 0.1% TFA) over 30 min at a flow rate of 1 ml min^{-1}. Detection was set to 215 nm. Identity of the peptide was confirmed by mass spectrometry.

Supplementary References

1. Miyata, K., Kawaguchi, M. & Nakagawa, T. Two distinct EIN2 genes cooperatively regulate ethylene signaling in lotus japonicus. *Plant and Cell Physiology* **54**, 1469-1477, (2013).
2. Ju, C. *et al.* Conservation of ethylene as a plant hormone over 450 million years of evolution. *Nature Plants* **1**, 1-7, (2015).
3. Edgar, R. C. MUSCLE: Multiple sequence alignment with high accuracy and high throughput. *Nucleic Acids Research* **32**, 1792-1797, (2004).
4. Tamura, K., Stecher, G., Peterson, D., Filipski, A. & Kumar, S. MEGA6: Molecular evolutionary genetics analysis version 6.0. *Molecular Biology and Evolution* **30**, 2725-2729, (2013).
